# Two faces of police stress: Spanish validation of operational and organizational PSQ scales

**DOI:** 10.3389/fpsyt.2026.1805061

**Published:** 2026-04-16

**Authors:** Susanna Rubiol Vilalta, Ana F. Moreno, Rowena Hill, Patrícia Oliveira-Silva

**Affiliations:** 1 Blanquerna Universitat Ramon Llull Facultat de Psicologia Ciencies de l’Educacio i de l’Esport, Barcelona, Spain; 2 Human Neurobehavioral Laboratory - HNL, Research Centre for Human Development - CEDH, Faculty of Education and Psychology, Universidade Católica Portuguesa - FEP-UCP, Porto, Portugal; 3 School of Social Sciences, Nottingham Trent University, Nottingham, United Kingdom

**Keywords:** Mossos d'esquadra, police, PSQ-Org, PSQ-OP, psychosocial risks, mental health

## Abstract

**Introduction:**

Police officers face multiple psychosocial risks stemming from operational and organizational aspects of their work. The Police Stress Questionnaire (PSQ) includes operational (PSQ-Op) and organizational (PSQ-Org) versions to assess these stressors. This study aimed to validate both versions in a sample of Mossos d’Esquadra, examining their factorial structure, reliability, and convergent and discriminant validity.

**Methods:**

Exploratory and confirmatory factor analyses were conducted to examine the internal structure of the PSQ-Op and PSQ-Org. Internal consistency was evaluated using reliability indices. Convergent and discriminant validity were assessed through correlations with measures of anxiety, depression, and coping strategies.

**Results:**

Both PSQ-Op and PSQ-Org showed an essentially unidimensional structure, indicating that each scale measures a coherent latent construct. Operational and organizational stress remained distinct domains. Both scales exhibited high reliability and adequate psychometric properties. Subtle gender differences were noted in the perception of specific stressors.

**Discussion:**

These findings support the validity and reliability of the PSQ-Op and PSQ-Org for assessing psychosocial risks among Spanish police officers. The scales can inform interventions targeting workplace stress prevention and the promotion of organizational well-being, emphasizing the need to address operational and organizational stressors separately.

## 1 Introduction

Police work is highly demanding, characterized by constant exposure to high-risk and high-responsibility situations, contributing to elevated stress levels that can affect physical and mental health, reduce performance, compromise decision-making, and deteriorate overall well-being (1–6). Occupational stress is multifactorial, determined by individual, organizational, and contextual factors, including emotional exhaustion, low perceived social support, inadequate coping strategies, high job demands, exposure to traumatic events, and organizational culture characteristics (7–12). Psychosocial factors at work have been formally recognized as key determinants of workers’ health and well-being (13).

From a theoretical perspective, stress constitutes a central construct in occupational health research, and in the policing context, it is mainly distinguished between operational stress and organizational stress, conceptually differentiable but interrelated domains (14–16). Operational stress refers to psychological, physiological, and behavioral responses to the demands inherent in operational activity, including intervention in violent incidents, exposure to traumatic events, decision-making under pressure, and constant threats to physical integrity (11, 17–19). In contrast, organizational stress emerges from the structure, roles, and processes of the organization and relates to role ambiguity and conflict, authoritarian leadership, bureaucratic pressure, rotating shifts, perceived injustice, and limited participation in decision-making (20–22).

### 1.1 Occupational stress: operational and organizational factors

Distinguishing between these types of stress is essential. Operational stress manifests at the micro level, associated with immediate demands and critical police tasks, such as emergency interventions or risk management. In contrast, organizational stress operates at the meso and macro levels: at the meso level, it arises from team dynamics, supervision, and intra-unit relationships; at the macro level, it relates to institutional culture, organizational policies, and management systems. Understanding this differentiation allows for more precise assessment of psychosocial risks and guides preventive interventions adapted to the policing context (14, 20). Nevertheless, both types of stress interact organizational stress can amplify the effects of operational stress, increasing vulnerability to critical incidents and the risk of negative consequences on mental health and performance (11, 19). This integrated perspective is essential for designing effective interventions that promote healthy organizational environments. In this regard, mindfulness-based interventions have shown positive effects on quality of life among Spanish police officers (23). However, the Spanish context is still lacking a measurement that allows for distinction of stressors derived from the police work, and the institutional factors that impact how police deal with those stressors.

#### 1.1.1 Operational stress subsection

Operational stress refers to stressors directly associated with police operational duties, including exposure to traumatic incidents, threats to personal safety, decision-making under pressure, and irregular working schedules. Research consistently shows that operational demands represent a central component of police stress and may contribute to fatigue, sleep disturbances, and psychological distress (11, 19). Instruments such as the Operational Police Stress Questionnaire (PSQ-Op) were developed to capture these stressors in a standardized manner and allow systematic monitoring of operational demands across policing contexts.

#### 1.1.2 Organizational stress subsection

Organizational stress refers to stressors arising from institutional structures and management practices within police organizations. These include bureaucratic procedures, leadership style, administrative workload, staffing shortages, and perceived organizational injustice (21, 22). Previous research has demonstrated that organizational stressors may be as influential as operational stressors in shaping police well-being and job satisfaction (11). The Organizational Police Stress Questionnaire (PSQ-Org) was specifically designed to capture these institutional stressors and to distinguish them from operational exposures.

### 1.2 Assessing occupational stress

Empirical evidence in Spain indicates that psychosocial risks, such as stress, have mainly been addressed and measured using generic instruments such as ISTAS21 (24), F-PSICO 4.0 (25), DECORE (26), and UNIPSICO (27), which evaluate general work organization conditions. Empirical evidence in Spain supports the role of psychosocial risk factors in predicting occupational well-being and stress outcomes (28, 29). These studies show consistent associations between workload, low control, role ambiguity, social support, and burnout symptoms (30–34). Other traditional stress instruments have also been used, such as the Post-Traumatic Stress Questionnaire (PCL), Stress Appreciation Scales (SEA), Perceived Stress Scale (PSS), Occupational Stress Inventory (OSI), and Maslach Burnout Inventory (MBI/MBI-GS), these focus on symptomatology and do not differentiate sources of stress or adequately capture the particularities of police work. Specific tools, such as the PSQ-Op and PSQ-Org, allow for a more comprehensive assessment but have not yet been validated in Spain (35, 36). While the PSQ has been validated in various cultural contexts, the absence of a Spanish police-specific validation has limited both systematic evaluation of stress from a psychosocial risk perspective and international comparability of occupational health results. This instrument is especially relevant for the police context as it allows differentiation between operational and organizational factors influencing police wellbeing. Among the operational factors, the scale addresses situations ranging from exposure to critical incidents to risk of physical harm and high**-**demand shifts, offering a comprehensive assessment of the typical demands faced by police officers. On the other hand, the organizational scale enables screening of the main challenges within police organizations, such as bureaucratic pressure, staffing shortages, and leadership challenges, providing valuable insights for targeted interventions and organizational improvements. While fairly recent, this measurement follows the wider literature that has differentiated these factors as relevant distinct influencing variables in the police psychological wellbeing (11; +3). Validating psychological scales requires consideration of internal consistency, as well as content, criterion, and construct validity, according to international standards of best practices (37, 38). Furthermore, cross-cultural adaptation processes require translation, back-translation, and pretesting to ensure semantic and conceptual equivalence across cultural contexts (39, 40). This study offers a validation and severity interpretation table for the Spanish police context, allowing to address a relevant gap in the occupational stress screening instruments available.

### 1.3 Research context and goals

Regarding the Spanish context, the Spanish public security system operates at three administrative levels. At the national level, the Guardia Civil and the National Police Corps have jurisdiction across the country, while regional forces, where Mossos d’Esquadra (Catalonia) are included, coexist and coordinate their actions with national forces. Local or municipal police operate in most municipalities, ensuring public safety, traffic control, and enforcement of local ordinances. This multi-level system is based on coordination and complementarity, as established by Organic Law 2/1986 on Security Forces and Corps (41). Thus, this study focuses on the Mossos d’Esquadra, Catalonia’s integrated police force, legally established under Law 10/1994 of the Police of the Generalitat – Mossos d’Esquadra (42), which implements the provisions of the 1979 Statute of Autonomy of Catalonia. This law provides the legal and institutional framework authorizing the Mossos d’Esquadra to operate as a regional force with responsibilities in public safety, public order, judicial and administrative policing, emergency assistance, inter-institutional cooperation, and protection of the environment, heritage, and public services. These functions are carried out under the principles of legality, coordination, and public service, ensuring citizens’ rights and freedoms, as well as the protection of people and property in Catalonia.

The Spanish legal framework for occupational risk prevention applicable to police forces is primarily based on Law 31/1995 on Occupational Risk Prevention, which transposes Directive 89/391/EEC, and is complemented by Royal Decree 39/1997, which approves the Regulation of Prevention Services (43), as well as by specific organic laws for each police body and guidelines on psychosocial risks. This framework establishes the obligation to assess both physical and psychosocial risks associated with each job position, implement preventive measures tailored to the particularities of police work, and ensure comprehensive protection of the physical and mental health of officers (Directive 89/391/EEC; 44, Law 31/1995; 45). Psychosocial risks are explicitly recognized as a legal obligation and must be evaluated and prevented to safeguard workers’ health, including members of police forces.–but this often does not translate into police and institutional practices, raising concerns about on-time assessment and intervention of police specific stressors and mental health.

Therefore, given the limitations of generic instruments and the lack of validated Spanish versions of the PSQ-Op and PSQ-Org, this study aims to (1) validate the PSQ-Op and PSQ-Org versions in the Spanish policing context; (2) analyze the factorial structure, reliability, and convergent and discriminant validity of both scales; and (3) explore gender differences in stress perception, to provide reliable and valid tools to assess psychosocial risk factors in police work, addressing a critical gap in occupational risk prevention among Spanish law enforcement personnel.

## 2 Methodology

### 2.1 Study design and procedure

A cross-sectional study design was employed, framed within the International Mental Health NeuroForce Project. The Police Stress Questionnaire (PSQ) was translated and culturally adapted into Spanish following internationally accepted guidelines for the cross-cultural adaptation of self-report instruments (40).

In the first phase, two independent bilingual translators performed direct translations of the original questionnaire into Spanish. Both versions were compared and synthesized into a single consensus version, ensuring semantic and conceptual equivalence of the items. Subsequently, this version underwent a back-translation process by two additional bilingual translators, independent from the original instrument, to assess conceptual equivalence between the original and translated versions.

To ensure contextual and terminological adequacy, four active police officers reviewed the final Spanish version and provided feedback on clarity, technical phrasing, and the relevance of the items within the policing context. Minor adjustments were made based on their observations. The language level of the final version was reviewed to ensure accessibility across educational backgrounds commonly found in police forces. The cross-cultural adaptation process followed internationally accepted guidelines for self-report measures. In addition to translation and back-translation, particular attention was given to semantic clarity, conceptual equivalence, and contextual relevance within the policing setting.

### 2.2 Participants

The final sample comprised 741 police officers from various operational units, predominantly male (75.0%), with a female representation of 24.8%, and a minority preferring not to identity their gender (*n*, 2). Most participants were married (61.8%), followed by those living with a partner (19.9%), while 10.9% were divorced and 7.0% single. Regarding educational level, the majority had completed high school (40.2%), followed by undergraduate or bachelor’s degrees (35.7%), with smaller proportions having completed secondary education (ESO, 9.6%) or a master’s degree (7.8%).

In terms of occupational characteristics, 68.9% of the sample had more than 10 years of service, primarily concentrated between 15 and 30 years of tenure. Most participants belonged to the basic rank (86.9%) and performed duties in Citizen Security (61.6%), followed by Investigation (22.1%), Traffic (9.3%), and Public Order (7.0%). More than half of the participants were active in the metropolitan area of Barcelona, primarily in the Metropolitana Nord (25.4%), Metropolitana de Barcelona (21.3%), and Metropolitana Sud (14.9%) regions.

### 2.3 Data collection

Data collection was conducted through an anonymous online survey, distributed via one of the main police unions in Catalonia between June and October 2023. Participation was voluntary, anonymous, and confidential. All participants were informed about the study objectives and provided informed consent before participation. The study protocol was approved by the relevant ethics committee and conducted in accordance with the principles of the Declaration of Helsinki and applicable data protection regulations.

wParticipants were recruited through one of the main police unions in Catalonia using an online survey format. This recruitment strategy was chosen to ensure broad and rapid dissemination across operational units while preserving anonymity. As participation was voluntary, the resulting sample constitutes a non-probabilistic sample.

### 2.4 Instruments

Police stress was assessed using the Police Stress Questionnaire (PSQ; 46), a 40-item self-report instrument designed to evaluate stress in police work through two dimensions: operational stress (PSQ- Op) and organizational stress (PSQ-Org). Items are rated on a Likert-type scale reflecting perceived stress intensity. For this study, the Spanish-adapted version specifically developed for this research was used.

Psychological symptomatology was measured using the Depression, Anxiety, and Stress Scales–21 (DASS-21; 47), consisting of 21 items rated on a 4-point Likert scale ranging from 0 (never) to 3 (almost always). The instrument evaluates symptoms of depression, anxiety, and stress. In the present sample, internal consistency was adequate for all subscales (depression, α, .88; anxiety, α, .83; stress, α, .83), consistent with previous studies in Spanish populations.

Coping strategies were assessed using the Brief COPE (48), a 28-item scale with a 4-point Likert response format (1, I haven’t been doing this at all; 4, I have been doing this a lot). The instrument evaluates 14 coping strategies and demonstrated acceptable overall internal consistency in this sample (α, .75), with reliability coefficients ranging from acceptable to very good across individual subscales.

### 2.5 Data analysis

Concerning data analysis, all sample groups with unrepresentative numbers (*n* < 10), specifically the group “Prefer not to say” (*n*, 2) were excluded from inferential analysis of gender comparisons, despite their inclusion for descriptive purposes only. Thus, for gender data analysis, the final sample retained was 739 following good statistical practices. Descriptive, correlational, and multivariate analyses were conducted to address the study objectives. The factorial structure of the instruments was examined using exploratory and confirmatory factor analyses, and reliability was assessed through internal consistency coefficients. Internal consistency was assessed using Cronbach’s alpha and additionally using McDonald’s omega calculated from standardized factor loadings obtained from unidimensional CFA models. Analyses were performed using IBM SPSS and AMOS (version 30.0). To address potential concerns regarding very high reliability values (α > 0.95), item redundancy and the scale’s bandwidth were examined, ensuring that items adequately captured the variability in perceived stress. This analysis confirmed that the high reliability reflected true internal consistency rather than excessive item overlap. Additionally, convergent and discriminant validity was assessed through AVE values and by comparing the square root of the AVE for each construct with the correlations among constructs, following the Fornell–Larcker criterion.

## 3 Results

### 3.1 Exploratory factor analysis

Before conducting exploratory (EFA) and confirmatory factor analyses (CFA), as well as reliability and convergent and discriminant validity analyses, the dataset was examined for missing values. Missing data were imputed to maintain statistical power and reduce potential bias (49). Following the recommendation of at least 10 participants per item for factor analysis, the sample was split into two subsets: 250 participants for the EFA and 491 for the CFA. Sample adequacy and data suitability for factor analysis were confirmed using the Kaiser-Meyer-Olkin (KMO) measure and Bartlett’s test of sphericity.

The suitability of the data for factor analysis was assessed using the Kaiser–Meyer–Olkin (KMO) measure of sampling adequacy and Bartlett’s test of sphericity. The results for both PSQ-Op and PSQ-Org indicated excellent sampling adequacy and statistically significant correlations among items (**Table 1**).

**Table 1:** Sampling adequacy tests for factor analysis.

Scale	KMO	Bartlett’s χ²	df	p
PSQ-Operational (PSQ-Op)	0.95	3986.72	190	<.001
PSQ-Organizational (PSQ-Org)	0.952	4942.9	190	<.001

KMO, Kaiser–Meyer–Olkin measure of sampling adequacy.

The EFA for the PSQ-Op demonstrated excellent suitability for factor analysis (KMO, .950; χ²(190), 3986.72, *p* <.001). Although three factors had eigenvalues greater than 1, the first factor accounted for 55.74% of the total variance, with a marked drop in subsequent eigenvalues, supporting the theoretical unidimensional structure (15, 46) (**Table 2**). All factor loadings were significant (≥.30) and communalities were adequate, with no evidence of multicollinearity. Internal consistency was excellent (α, .958; ω, .94), and inter-item correlations confirmed item homogeneity (mean r, .71, range, .52–.82), with an average inter-item correlation of.49 indicating high consistency without evidence of problematic redundancy.

**Table 2:** Exploratory factor analysis of the operational police stress questionnaire (PSQ-Op).

Item	Description	*M* (SD)	2-factor extraction			1-factor extraction	
			*h*²	F1	F2	*h*²	F1
1	*Shift work*	3.34 (2.03)	0.66	**0.91**	0.14	0.53	0.73
2	*Working alone at night*	3.29 (2.21)	0.57	**0.86**	0.15	0.45	0.67
12	*Fatigue (e.g., shift work, over-time)*	4.06 (2.27)	0.76	**0.81**	-0.08	0.70	**0.84**
4	*Risk of being injured on the job*	3.05 (1.84)	0.49	**0.73**	0.04	0.42	0.65
13	*Occupation-related health issues (e.g., back pain)*	4.16 (2.18)	0.64	**0.70**	-0.12	0.61	0.78
6	*Traumatic Events (e.g., MVA, domestics death, injury)*	3.34 (1.99)	0.51	**0.70**	-0.02	0.46	0.68
5	*Work-related activities on days off (e.g., court, community events)*	3.18 (1.91)	0.53	**0.61**	-0.15	0.51	0.71
8	*Not enough time available to* sp*end with friends and family*	3.51 (2.14)	0.62	**0.60**	-0.23	0.61	0.78
11	*Finding time to stay in good physical condition*	3.80 (1.07)	0.59	**0.58**	-0.23	0.58	0.76
10	*Eating healthy at work*	3.22 (1.97)	0.47	**0.57**	-0.14	0.45	0.67
3	*Over-time demands*	2.35 (1.76)	0.31	**0.52**	-0.05	0.28	0.53
7	*Managing your social life outside of work*	3.22 (1.96)	0.55	**0.42**	-0.37	0.55	0.74
9	*Paperwork*	3.64 (2.01)	0.40	**0.41**	-0.27	0.41	0.64
16	*Upholding a “higher image” in public*	2.72 (1.85)	0.82	-0.14	**-1.01**	0.60	0.77
18	*Limitations to your social life (e.g., who your friends are, where you socialize)*	2.99 (1.93)	0.76	0.06	**-0.83**	0.65	0.81
17	*Negative comments from the public*	3.36 (2.06)	0.69	0.05	**-0.80**	0.59	0.77
20	*Friends/family feel the effects of the stigma associated with your job*	2.76 (1.90)	0.67	0.04	**-0.79**	0.57	0.76
15	*Making friends outside the job*	2.60 (1.85)	0.59	0.05	**-0.73**	0.51	0.72
19	*Feeling like you are always on the job*	3.62 (2.04)	0.68	0.21	**-0.66**	0.64	0.80
14	*Lack of understanding from family and friends about your work*	3.08 (2.03)	0.62	0.17	**-0.65**	0.58	0.76
**Cronbach’s Alpha**				0.937	0.938		0.958

*N*, 250; communality, *h2;* Factor - F. Extraction method: principal axis factoring (PAF) with oblique rotation. Factor loadings above.30 are shown in bold for 2-factor extraction. Cross loadings are underlined. PSQ-Op, Operational Police Stress Questionnaire.

The EFA for the PSQ-Org also demonstrated high suitability for factor analysis (KMO, .952; χ²(190), 4942.90, *p* <.001). The first factor accounted for 62.6% of the total variance, and both parallel analysis and the scree plot supported a unidimensional structure. Internal consistency was likewise excellent (α, .968; ω, .96), with mean inter-item correlations of .76 (range, .61–.85), confirming construct coherence (**Table 3**), with an average inter-item correlation of.56 indicating no evidence for problematic redundancy.

**Table 3:** Exploratory factor analysis of the organizational police stress questionnaire (PSQ-Org).

Item.	Description		*M (SD)*		2-factor extraction		1-factor extraction	
				*h*²	F1	F2	*h*²	F1
7	*Bureaucratic red tape*		4.06 (2.11)	0.78	**0.94**	-0.07	0.68	0.82
9	*Lack of training on new equipment*		4.00 (2.06)	0.77	**0.90**	-0.03	0.69	0.83
20	*Inadequate equipment*		4.21 (2.14)	0.72	**0.88**	-0.04	0.64	0.80
8	*Too much computer work*		3.48 (2.08)	0.66	**0.87**	-0.08	0.58	0.76
5	*Constant changes in policy/legislation*		3.83 (2.10)	0.75	**0.83**	0.04	0.70	0.83
4	*Excessive administrative duties*		3.91 (2.10)	0.68	**0.81**	0.02	0.62	0.79
13	*Lack of resources*		4.57 (2.08)	0.74	**0.76**	0.13	0.71	0.84
6	*Staff shortages*		4.68 (2.09)	0.76	**0.70**	0.21	0.75	**0.87**
18	*Dealing the court system*		3.22 (2.12)	0.51	**0.61**	0.13	0.492	0.70
10	*Perceived pressure to volunteer free time*		3.33 (2.01)	0.63	**0.51**	0.33	0.628	0.79
19	*The need to be accountable for doing your job*		3.59 (2.24)	0.71	**0.474**	0.42	0.71	0.84
17	*Internal investigations*		2.83 (2.14)	0.42	**0.349**	0.342	0.42	0.65
16	*Leaders over-emphasize the negatives (e.g. supervisor evaluations, public complaints)*		3.31 (2.15)	0.67	-0.03	**0.84**	0.55	0.74
15	*If you are sick or injured your co-workers seem to look down on you*		2.62 (1.97)	0.55	-0.12	**0.83**	0.41	0.64
2	*The feeling that different rules apply to different people (e.g. favoritism)*		3.88 (2.14)	0.68	0.01	**0.82**	0.57	0.76
11	*Dealing with supervisors*		3.29 (2.02)	0.73	0.05	**0.81**	0.63	0.79
1	*Dealing with coworkers*		2.95 (1.81)	0.49	-0.08	**0.76**	0.39	0.62
12	*Inconsistent leadership style*		3.50 (2.08)	0.66	0.14	**0.70**	0.60	0.78
3	*Feeling like you always have to prove yourself to the organization*		3.78 (2.10)	0.69	0.20	**0.66**	0.65	0.80
14	*Unequal sharing of work responsibilities*		4.12 (2.18)	0.75	0.33	**0.59**	0.74	0.86
**Cronbach’s Alpha**					0.958	0.925		0.968

*N*, 250; Communality, *h2;* Factor - F. Extraction method: principal axis factoring (PAF) with oblique rotation. Factor loadings above.30 are shown in bold for 2-factor extraction. Crossloadings are underlined. PSQ-Org, Organizational Police Stress Questionnaire.

### 3.2 Confirmatory factor analysis

Confirmatory factor analyses (CFA) evaluated three models for each scale: unidimensional, correlated two-factor, and second order. For both instruments, all models demonstrated adequate fit; however, the unidimensional model was retained due to its parsimony and theoretical coherence.

The final model selection was not based solely on “fit” but on multiple fit indices (CFI ≥ 0.95, TLI ≥ 0.95, RMSEA ≤ 0.06, SRMR ≤ 0.08) in accordance with best practices for psychometric scale validation (50–52). The unidimensional model was preferred for its simplicity and theoretical consistency, as alternative models (two-factor and second order) did not offer practically relevant improvements. All results were interpreted strictly based on the observed data, avoiding extrapolations regarding mental health or performance that the analyses did not support. This approach ensures methodological rigor consistent with international standards for scale validation (53, 54).

For the PSQ-Op (**Table 4**), the unidimensional model demonstrated excellent fit (χ²(61), 65.56, p, .322; CFI, .999; TLI, .998; RMSEA, .012; SRMR, .017). For the PSQ-Org (**Table 4**), similarly excellent fit was observed (χ²(59), 65.65, p, .257; CFI, .999; TLI, .997; RMSEA, .015; SRMR, .014). The two-factor and second-order models showed only marginal differences in parsimony and complexity without practical relevance, confirming the unidimensional model as the optimal representation of the perceived stress construct. PSQ-Org followed the same trend, with all models demonstrating acceptable to excellent fit. However, the unidimensional model exhibited the strongest fit indices (χ² (59), 65.65, p, .257; CFI, .999; TLI, .997; RMSEA, .015; SRMR, .014; AIC, 407.65; ECVI, 0.83), consistent with the theoretical expectation of a single underlying construct. The two-factor and higher order models showed good fit with marginal differences as well, however showing higher model complexity or less favorable parsimony indices. All models indicated acceptable stability (Hoelter’s N (0.05) > 200).

**Table 4:** Comparison of confirmatory factor analysis models for the PSQ-Op and PSQ-Org.

Model	χ²(df), *p*	Comparative fit indices			Absolute Fit indices			Parsimony-adjusted fit indices	
		CFI	TLI	IFI	RMSEA	PCLOSE	SRMR	AIC	ECVI
					[90% CI]				
PSQ-Op									
*Unidimensional Model*	65.56 (61),.322	0.999	0.998	0.999	.012	1.00	0.017	403.56	0.82
					[.000–.031]				
*2-Factor* *Model*	92.37 (83),.226	0.998	0.997	0.999	.015	1.00	0.020	386.37	0.79
					[.000–.030]				
*Higher-Order 2-Factor Model*	87.27 (80),.271	0.999	0.997	0.999	.014	1.00	0.020	387.27	0.79
					[.000–.030]				
PSQ-Org									
*Unidimensional Model*	65.65 (59),.257	0.999	0.997	0.999	.015	1.00	0.014	407.65	0.83
					[.000–.033]				
*2-Factor* *Model*	95.53 (76),.064	0.998	0.994	0.998.	.023	1.00	0.021	403.53	0.82
					[.000–.036]				
*Higher-Order 2-Factor Model*	106.99 (78),.016	0.996	0.991	0.996	.028	1.00	0.022	410.99	0.84
					[.012–.040]				

*N*, 491; CFI, Comparative Fit Index. TLI, Tucker–Lewis Index; RMSEA, Root Mean Square Error of Approximation; SRMR, Standardized Root Mean Square Residual; AIC, Akaike Information Criterion; ECVI, Expected Cross-Validation Index. Lower AIC and ECVI values indicate greater parsimony. All models demonstrated excellent global fit. RMSEA values below.05 and CFI/TLI above.95 indicate excellent fit. PCLOSE tests the hypothesis that RMSEA is ≤.05.

Given that the instrument was originally developed as unidimensional (15, 46), and that the unidimensional model demonstrated fit indices meeting or exceeding conventional thresholds for excellent fit, it was retained as the most appropriate and parsimonious solution. This decision prioritizes theoretical consistency and interpretability while ensuring strong empirical support, as evidenced by multiple fit indices, including a particularly low SRMR (.017), reflecting minimal residual misfit. Accordingly, the unidimensional model was selected as the optimal representation of the construct. These results support an essentially unidimensional internal structure for each questionnaire, indicating coherent measurement within each domain. However, the observed unidimensionality does not imply the existence of a single global stress factor but rather reflects the internal consistency of two related yet conceptually distinct constructs.

### 3.3 Convergent and divergent validity

Convergent validity was evidenced by moderate to strong correlations with the stress subscale of the DASS-21 (PSQ-Op: *r*, .514; PSQ-Org: *r*, .513; *p* <.001) and by average variance extracted (AVE) values above.50 (PSQ-Op, .53; PSQ-Org, .61) (**Table 4**). Divergent validity was confirmed by comparing squared correlations with the AVE values: all correlations with the subconstructs of depression, anxiety, and coping strategies from the Brief COPE were lower than the corresponding AVE, indicating that each dimension shares more variance with its own items than with theoretically unrelated constructs, thus supporting discriminant validity. Discriminant validity was further examined using the Fornell–Larcker criterion, through comparison of the square root of the AVE for each construct. The PSQ dimensions showed adequate discriminant validity in relation to external constructs, including DASS-21 stress, depression, and anxiety, as well as coping dimensions, as correlations with these variables were lower than the square root of their AVE values. However, discriminant validity between PSQ-Op and PSQ-Org themselves was not supported by the Fornell–Larcker criterion as expected, given the strong association between the two dimensions as two police stress occupational facets (**Table 4**). Although the Fornell–Larcker criterion was not fully satisfied between PSQ-Op and PSQ-Org, this result is theoretically expected because both dimensions represent closely related facets of occupational police stress. 

### 3.4 Cut-off scores for Mossos d’Esquadra population

Stress levels were established by dividing normative scores into tertiles (15) (**Table 5**). For the PSQ-Op, the ranges in this sample were: low < 2.40, moderate 2.40–4.10, and high > 4.10; for the PSQ-Org, the ranges were: low < 2.70, moderate 2.70–4.70, and high > 4.70. The mean stress levels observed in the sample (**Table 6**) fell within the moderate range for both operational stress (*M*, 3.30, *SD*, 1.40) and organizational stress (*M*, 3.74, *SD*, 1.56), indicating that most participants perceive stress at a moderate intensity, although considerable individual variability was present.

**Table 5:** Fornell–Larcker discriminant validity matrix.

Variable	1.	2.	3.	4.	5.	6.	7.	8.
1. PSQ-Operational (PSQ-Op)	**.73**							
2. PSQ-Organizational (PSQ-Org)	.81	**.78**						
3. DASS-21 Stress	.52	.52	**.73**					
4. DASS-21 Depression	.43	.44	.79	**.79**				
5. DASS-21 Anxiety	.40	.41	.77	.77	**.71**			
6. Problem-Focused Coping	.03	.05	.03	-.06	.00	**.86**		
7. Emotion-Focused Coping	.12	.11	.17	.11	.13	.65	**.71**	
8. Avoidant Coping	.26	.27	.44	.48	.46	.14	.39	.**64**

*N*, 741; Two-tailed Pearson coefficients are depicted. Diagonals represented in bold refer to the square root of the average variance extracted (AVE). PSQ-Op, Operational Police Stress Questionnaire; PSQ-Org, Organizational Police Stress Questionnaire; DASS, Depression Anxiety Stress Scales. All correlations are significant at *p* <.001.

Correlation between police stress questionnaire scores and DASS-21 subscales.

**Table 6:** Sample-based stress level classification for the PSQ-Op and PSQ-Org.

	Low stress	Moderate stress	High stress
PSQ-Op	< 2.40	2.40-4.10	> 4.10
PSQ-Org	< 2.70	2.70-4.70	> 4.70

Stress categories were derived using tertile splits of the sample distribution, following the approach proposed by McCreary et al. (15). These ranges are sample-specific and should not be interpreted as population norms.

Gender-based analyses (**Table 7**) revealed slightly higher mean operational stress levels among men. For PSQ-Op, men reported marginally higher scores (*M*, 3.36, *SD*, 1.38) compared to women (*M*, 3.16, *SD*, 1.45). Similarly, for PSQ-Org, men also showed slightly higher scores (*M*, 3.80, *SD*, 1.55) than women (*M*, 3.58, *SD*, 1.56). These differences were primarily observed in items related to physical and administrative demands, whereas women reported comparable levels on items associated with fatigue and occupational health. However, none of these differences reached statistical significance (*p* >.05) and are therefore presented only descriptively.

**Table 7:** Descriptive statistics for PSQ-Op and PSQ-Org by gender.

Summary Score	Male (n, 557)	Female (n, 182)	Total (N, 739)
PSQ-Op			
Mean (SD)	3.36 (1.38)	3.16 (1.45)	3.30 (1.40)
95% CI	3.24–3.47	2.95–3.37	3.21-3.41
PSQ-Org			
Mean (SD)	3.80 (1.55)	3.58 (1.56)	3.74 (1.56)
95% CI	3.67–3.93	3.35–3.81	3.63-3.86

*N*, 739; The gender class “Prefer not to say” was removed due to low representativeness (n=2). PSQ-Op, Operational Police Stress Questionnaire; PSQ-Org, Organizational Police Stress Questionnaire; *SD*, Standard-Deviation; CI, Confidence Interva.l

Item-level analyses of the PSQ-Op indicated that men perceived greater stress associated with direct job demands and administrative tasks, such as shift work (3.52 vs. 3.31), working alone at night (3.49 vs. 3.27), and paperwork load (3.70 vs. 3.29) (**Appendix 1**). In contrast, women reported comparable stress levels on items related to fatigue (4.12 vs. 4.16) and occupational health issues (4.31 vs. 4.28), suggesting that, although overall differences are small, the perception of specific operational demands may vary by gender (**Appendix 1**).

Regarding organizational stress (PSQ-Org), men perceived greater pressure from structural and bureaucratic aspects of the organization, including lack of resources (4.82 vs. 4.62), internal bureaucracy (4.38 vs. 3.87), and frequent policy changes (4.05 vs. 3.53) (**Appendix 2**). Women, on the other hand, reported similar stress levels concerning the need to demonstrate competence within the organization (3.78 vs. 3.70) and interactions with supervisors, indicating that organizational stress manifests similarly across genders, albeit with emphasis on different types of demands (**Appendix 2**). These findings highlight the importance of considering both stress type and gender differences; they should not be interpreted as evidence of true gender disparities, but rather as useful information for future research or more targeted explorations.

Overall, these results confirm the validity and reliability of the PSQ scales, supporting their use in measuring both operational and organizational police stress. They facilitate interpretation at both the global scale and item level, as well as the identification of individuals with moderate to high stress levels for intervention or monitoring purposes.

## 4 Discussion

The present study aimed to validate the Spanish versions of the Operational (PSQ-Op) and Organizational (PSQ-Org) Police Stress Questionnaires. Results confirmed their factorial structure, internal consistency, and convergent and discriminant validity, supporting their use in assessing distinct facets of occupational stress among Spanish police officers. These findings are consistent with prior research in other contexts (35, 46) and provide a practical tool for targeted interventions to reduce police stress.

### 4.1 Psychometric structure and conceptual interpretation

Both exploratory and confirmatory factor analyses supported an essentially unidimensional internal structure within each scale, indicating that the PSQ-Op and PSQ-Org each assess a coherent latent construct. Importantly, this unidimensionality applies within scales and does not imply the existence of a single global stress factor. Rather, the findings are fully consistent with the original conceptualization of police stress as comprising two distinct but interrelated domains, operational and organizational stress (15, 55).

The strong correlation observed between PSQ-Op and PSQ-Org scores reflects the empirical interdependence between operational demands and organizational conditions in policing, a relationship well documented in the literature (11, 14, 19). Organizational stressors such as bureaucratic pressure, staffing shortages and leadership practices may intensify the impact of operational stress by constraining recovery, autonomy and perceived support, thereby increasing overall vulnerability to stress-related outcomes. From a psychometric standpoint, confirming internal unidimensionality within each scale enhances interpretability and usability, while preserve the analytical distinction required for targeted assessment and intervention.

### 4.2 Reliability and construct validity

Both PSQ-Op and PSQ-Org demonstrated excellent internal consistency, with Cronbach’s alpha and McDonald’s omega coefficients exceeding conventional thresholds. Although very high reliability values warrant consideration of potential item redundancy (53), inter-item correlation patterns and item-level analyses indicated adequate content breadth and variability, suggesting that the observed reliability reflects true construct coherence rather than excessive overlap. These findings are consistent with previous PSQ validations conducted in other cultural contexts (15, 56).

Convergent validity was supported by moderate to strong correlations with the stress subscale of the DASS-21 (47), while discriminant validity was evidenced by weaker associations with depression, anxiety, and coping strategies, as well as by satisfactory average variance extracted values (37, 38). Together, these results indicate that the PSQ captures stressors specific to police work rather than general psychological distress, reinforcing its added value relative to symptom-focused measures such as the DASS-21, PSS, or burnout inventories commonly used in Spanish samples (30, 31, 34). Stress levels and interpretative scope.

Mean levels of operational and organizational stress in this sample fell within the moderate range when classified using sample-adapted intervals derived from tertile splits, following the approach proposed by McCreary et al. (15). This pattern aligns with prior evidence indicating that most police officers report moderate stress levels, while a non-negligible proportion experience elevated stress that may increase risk for adverse mental health and occupational outcomes (8, 11).

The use of sample-based classification intervals allows for a nuanced description of stress distribution within the studied population but should be interpreted as descriptive rather than normative. These thresholds do not constitute population-level cut-offs and should not be equated with clinical or epidemiological prevalence estimates. Nonetheless, the capacity of the PSQ to differentiate levels of perceived stress across the workforce is particularly relevant for occupational risk prevention frameworks, which emphasize early identification and primary prevention rather than reactive intervention (25, 57).

### 4.3 Gender patterns in stress perception

Gender-based analyses revealed broadly comparable levels of operational and organizational stress among men and women, with only subtle, non-significant differences observed at the item level. Men tended to report slightly higher stress related to physical demands, administrative burden, and organizational constraints, whereas women reported comparable levels of fatigue and health-related stressors. Similar patterns have been reported in previous Spanish and international studies, where gender differences in police stress are generally modest and context-dependent (9, 33).

Given the absence of statistically significant differences and the lack of formal measurement invariance testing, these findings should be interpreted descriptively. Rather than indicating true gender disparities, they suggest that stressors may be perceived or articulated differently depending on role expectations and organizational positioning. This underscores the importance of incorporating a nuanced, context-sensitive perspective when designing psychosocial risk prevention strategies, in line with current occupational health recommendations (25).

### 4.4 Implications for occupational risk prevention and practice

The validation of the PSQ-Op and PSQ-Org in Spain has direct and practical implications for occupational risk prevention in police forces. Spanish legislation explicitly mandates the assessment and management of psychosocial risks (Law 31/1995; RD 39/1997; Directive 89/391/EEC). However, police organizations have traditionally relied on generic instruments that may fail to capture the specific stressors inherent to policing. The PSQ provides a police-specific, psychometrically robust tool that enables a clear differentiation between stress arising from operational exposure and stress rooted in organizational structures and processes.

This distinction is critical for effective intervention design. Operational stressors may be addressed through training, peer support, recovery-oriented strategies and exposure management, whereas organizational stressors require structural and managerial responses, including workload redistribution, administrative burden optimization, leadership development and procedural reform (14, 17, 22). By allowing separate yet complementary assessment of these domains, the PSQ supports a more precise, targeted and actionable approach to stress prevention and organizational well-being promotion.

In addition, the use of sample-based classification intervals enables organizations to estimate the proportion of officers experiencing moderate or high stress, facilitating prioritization of resources and early preventive action. Although observed gender differences did not reach statistical significance and should therefore be interpreted cautiously, item-level patterns suggest that stressors may be perceived differently across subgroups. This underscores the importance of context-sensitive prevention strategies that consider organizational, cultural and role-related factors rather than relying on uniform or demographically prescriptive approaches. Systematic implementation of such evidence-informed measures has the potential to enhance officers’ well-being, motivation, and performance, contributing to the long-term sustainability and effectiveness of public safety organizations.

### 4.5 Limitations

First, the sample was obtained through voluntary participation via a police union and an online questionnaire, which may introduce self-selection bias. Officers experiencing higher levels of stress or greater interest in psychosocial issues may have been more likely to participate. Consequently, the sample cannot be considered probabilistic, and the findings should be interpreted with caution regarding population-level prevalence estimates. Nevertheless, the demographic and occupational characteristics of the sample (sex distribution, rank, years of service, and operational units) broadly reflect the known structure of the Mossos d’Esquadra workforce, supporting the relevance of the sample for psychometric validation purposes. Also, because the survey was disseminated through union mailing lists, an exact response rate could not be calculated.

Second, although Catalonia is a bilingual context (Spanish and Catalan), the present study used a Spanish version of the PSQ. While Spanish is widely used in professional and administrative settings within the Mossos d’Esquadra, subtle linguistic or cultural nuances may influence item interpretation. Future research should therefore examine the psychometric equivalence of a Catalan version and formally test linguistic measurement invariance.

Although the instruments demonstrated high reliability, their temporal stability should be explored through longitudinal studies. Furthermore, the stress intervals used were adapted to the sample distribution via tertile splits of normative scores and cannot be considered official standards for the Spanish population. Generalization of these thresholds requires further research with representative samples and formal validation of the instruments across diverse police contexts.

Addressing these methodological aspects in future studies will strengthen the generalizability and cross-cultural robustness of the PSQ in multilingual policing contexts.

## 5 Future research directions

Future research should focus on validating the PSQ-Op and PSQ-Org in other Spanish and international police forces, considering cultural, organizational, and legal differences that may influence stress perception. Longitudinal studies exploring the relationship between operational and organizational stressors and physical and mental health outcomes, job performance, and staff turnover are warranted. Research integrating gender and other sociodemographic factors could deepen understanding of how individual and social differences modulate stress experiences. Finally, the effectiveness of preventive interventions and psychological support programs specifically designed for police officers should be investigated to establish evidence-based strategies that enhance well-being and resilience.

In conclusion, the present study validated the Spanish PSQ-Op and PSQ-Org scales, confirming their factorial structure, reliability, and convergent and discriminant validity. These instruments provide a practical and psychometrically robust tool for assessing occupational stress among Spanish police officers, enabling targeted interventions for both operational and organizational stressors. Implementation of the PSQ can support evidence-based occupational risk prevention, enhance well-being, and inform organizational practices within law enforcement contexts.

## Data Availability

The raw data supporting the conclusions of this article will be made available by the authors, without undue reservation.
