# Supplementary material for: Two faces of police stress: Spanish validation of operational and organizational PSQ scales
Source: Front Psychiatry. 2026 Apr 16;17:1805061. doi: 10.3389/fpsyt.2026.1805061 (PMC13128590; doi:10.3389/fpsyt.2026.1805061)
Supplement: Supplementary file 1 [file DataSheet1.pdf]

Appendix 1. Item-Level Descriptive Statistics for the PSQ-Op by Gender

Scale item	Male (n = 557)	Female (n = 182)	Both genders
1. Shift work			
Mean (*SD*)	3.52 (2.00)	3.31 (2.04)	3.47 (2.01)
95% CI	3.36–3.69	3.01–3.61	3.32–3.61
2. Working alone at night			
Mean (*SD*)	3.49 (2.28)	3.27 (2.19)	3.43 (2.26)
95% CI	3.30–3.68	2.95–3.59	3.27–3.60
3. Overtime demands			
Mean (*SD*)	2.44 (1.83)	2.37 (1.76)	2.42 (1.81)
95% CI	2.29–2.59	2.12–2.63	2.29–2.55
4. Risk of being injured on the job			
Mean (*SD*)	3.34 (1.85)	3.07 (1.71)	3.27 (1.82)
95% CI	3.18–3.49	2.82–3.32	3.14–3.40
5. Work-related activities on days off			
Mean (*SD*)	3.33 (1.88)	3.07 (1.88)	3.27 (1.89)
95% CI	3.18–3.49	2.79–3.34	3.13–3.40
6. Traumatic Events (e.g., MVA, domestic death, injury)			
Mean (*SD*)	3.50 (1.98)	3.25 (1.98)	3.43 (1.98)
95% CI	3.33–3.66	2.96–3.54	3.29–3.58
7. Managing your social life outside work			
Mean (*SD*)	3.33 (1.93)	3.26 (2.06)	3.31 (1.96)
95% CI	3.17–3.49	2.96–3.56	3.17–3.45
8. Not enough time available to spend with friends and family			
Mean (*SD*)	3.58 (2.00)	3.62 (2.10)	3.59 (2.03)
95% CI	3.41–3.74	3.31–3.92	3.44–3.73
9. Paperwork			
Mean (*SD*)	3.70 (2.01)	3.29 (2.02)	3.60 (2.02)
95% CI	3.53–3.86	2.99–3.58	3.45–3.74
10. Eating healthy at work			
Mean (*SD*)	3.44 (1.95)	3.10 (1.96)	3.36 (1.95)
95% CI	3.28–3.61	2.81–3.38	3.22–3.50
11. Finding time to maintain good physical condition			
Mean (*SD*)	3.82 (2.03)	3.75 (2.02)	3.80 (2.02)
95% CI	3.65–3.99	3.45–4.04	3.66–3.95
12. Fatigue			
Mean (*SD*)	4.16 (2.10)	4.12 (2.14)	4.15 (2.11)
95% CI	3.99–4.34	3.80–4.43	4.00–4.30
13. Occupation-related health issues			
Mean (*SD*)	4.28 (2.08)	4.31 (2.09)	4.29 (2.08)
95% CI	4.11–4.45	4.00–4.61	4.14–4.44
14. Lack of understanding from family and friends			
Mean (*SD*)	3.10 (1.98)	2.84 (1.95)	3.04 (1.97)
95% CI	2.94–3.27	2.55–3.12	2.89–3.18
15. Difficulty making friends outside work			
Mean (*SD*)	2.50 (1.73)	2.54 (1.85)	2.51 (1.76)

95% CI	2.35–2.64	2.27–2.81	2.38–2.63
16. Upholding a ‘higher image’ in public			
Mean (*SD*)	2.66 (1.77)	2.37 (1.77)	2.59 (1.78)
95% CI	2.51–2.81	2.12–2.63	2.46–2.72
17. Negative comments from the public			
Mean (*SD*)	3.45 (2.01)	3.14 (1.98)	3.37 (2.01)
95% CI	3.28–3.61	2.85–3.43	3.23–3.52
18. Limitations to your social life			
Mean (*SD*)	2.97 (1.83)	2.75 (1.86)	2.91 (1.84)
95% CI	2.81–3.12	2.48–3.02	2.78–3.05
19. Feeling like you are always on the job			
Mean (*SD*)	3.70 (2.03)	3.25 (2.04)	3.59 (2.04)
95% CI	3.53–3.87	2.95–3.55	3.44–3.73
20. Friends/ family feel the effect of the stigma associated with your job			
Mean (*SD*)	2.86 (1.89)	2.56 (1.81)	2.79 (1.87)
95% CI	2.71–3.02	2.30–2.83	2.65–2.92

Note. N= 739; PSQ-Op = Operational Police Stress Questionnaire; *SD* = Standard-Deviation; CI = Confidence Interval
