# Supplementary material for: Two faces of police stress: Spanish validation of operational and organizational PSQ scales
Source: Front Psychiatry. 2026 Apr 16;17:1805061. doi: 10.3389/fpsyt.2026.1805061 (PMC13128590; doi:10.3389/fpsyt.2026.1805061)
Supplement: Supplementary file 2 [file DataSheet2.pdf]

Appendix 2. Item-Level Descriptive Statistics for the PSQ-Org by Gender

Scale item	Male (n = 557)	Female (n = 182)	Both genders
1. Dealing with co-workers			
Mean (SD)	2.92 (1.74)	2.88 (1.83)	2.91 (1.76)
95% CI	2.78–3.07	2.61–3.15	2.79–3.04
2. The feeling that different rules apply to different people			
Mean (SD)	3.97 (2.09)	3.80 (2.04)	3.93 (2.08)
95% CI	3.80–4.15	3.50–4.09	3.78–4.08
3. Feeling like you always have to prove yourself to the organization			
Mean (SD)	3.70 (1.96)	3.78 (2.06)	3.72 (1.99)
95% CI	3.53–3.86	3.48–4.08	3.57–3.86
4. Excessive administrative duties			
Mean (SD)	4.09 (2.06)	3.64 (2.12)	3.98 (2.08)
95% CI	3.92–4.26	3.33–3.95	3.83–4.13
5. Constant changes in policy/legislation			
Mean (SD)	4.05 (2.01)	3.53 (2.06)	3.92 (2.03)
95% CI	3.88–4.22	3.23–3.83	3.77–4.07
6. Staff shortages			
Mean (SD)	4.99 (2.00)	4.81 (2.04)	4.94 (2.01)
95% CI	4.82–5.15	4.52–5.11	4.80–5.09
7. Bureaucratic red tape			
Mean (SD)	4.38 (2.01)	3.87 (2.09)	4.26 (2.04)
95% CI	4.22–4.55	3.56–4.17	4.11–4.40
8. Too much computer work			
Mean (SD)	3.64 (2.02)	3.59 (2.18)	3.62 (2.06)
95% CI	3.47–3.80	3.27–3.91	3.47–3.77
9. Lack of training on new equipment			
Mean (SD)	4.24 (2.02)	4.10 (2.01)	4.20 (2.02)
95% CI	4.07–4.40	3.81–4.40	4.06–4.35
10. Perceived pressure to volunteer free time			
Mean (SD)	3.43 (1.94)	3.30 (2.03)	3.40 (1.96)
95% CI	3.27–3.59	3.00–3.59	3.25–3.54
11. Dealing with supervisors			
Mean (SD)	3.33 (1.95)	3.12 (1.97)	3.28 (1.96)
95% CI	3.17–3.49	2.83–3.40	3.13–3.42
12. Inconsistent leadership style			
Mean (SD)	3.53 (2.05)	3.37 (2.13)	3.49 (2.07)
95% CI	3.36–3.70	3.06–3.68	3.34–3.64
13. Lack of resources			
Mean (SD)	4.82 (2.01)	4.62 (2.09)	4.77 (2.03)
95% CI	4.66–4.99	4.31–4.92	4.63–4.92
14. Unequal sharing of work responsibilities			
Mean (SD)	4.24 (2.17)	4.10 (2.25)	4.20 (2.19)
95% CI	4.05–4.42	3.77–4.43	4.04–4.36
15. If you are sick or injured your coworkers seem to look down on you			
Mean (SD)	2.60 (1.93)	2.68 (2.06)	2.62 (1.96)

95% CI	2.44–2.76	2.38–2.98	2.48–2.76
16. Leaders overemphasize the negatives			
Mean (SD)	3.36 (2.14)	3.27 (2.14)	3.34 (2.14)
95% CI	3.18–3.54	2.96–3.59	3.19–3.50
17. Internal investigations			
Mean (SD)	3.10 (2.18)	2.45 (1.77)	2.94 (2.10)
95% CI	2.91–3.28	2.19–2.71	2.78–3.09
18. Dealing with the court system			
Mean (SD)	3.46 (2.00)	2.91 (1.85)	3.32 (1.98)
95% CI	3.29–3.63	2.64–3.18	3.18–3.47
19. The need to be accountable for doing your job			
Mean (SD)	3.68 (2.15)	3.55 (2.12)	3.65 (2.14)
95% CI	3.50–3.86	3.24–3.86	3.49–3.80
20. Inadequate equipment			
Mean (SD)	4.46 (2.08)	4.30 (2.15)	4.42 (2.10)
95% CI	4.29–4.64	3.99–4.62	4.27–4.57

Note. N=739; PSQ-Org = Organizational Police Stress Questionnaire; *SD* = Standard-Deviation; CI = Confidence Interval
